# The Management of Prodromal Symptoms of Bipolar Disorder: Available Options and Future Perspectives

**DOI:** 10.3390/medicina57060545

**Published:** 2021-05-28

**Authors:** Elisa Del Favero, Cristiana Montemagni, Paola Bozzatello, Claudio Brasso, Cecilia Riccardi, Paola Rocca

**Affiliations:** 1 Dipartimento di Neuroscienze “Rita Levi Montalcini”, Università degli Studi di Torino, Via Cherasco 11, 10126 Turin, Italy; elisa.delfavero@unito.it (E.D.F.); paola.bozzatello@unito.it (P.B.); claudio.brasso@unito.it (C.B.); cecilia.riccardi@unito.it (C.R.); 2 Dipartimento di Neuroscienze e Salute Mentale, A.O.U. Città Della Salute e Della Scienza, via Cherasco 11, 10126 Turin, Italy; cristiana.montemagni@unito.it

**Keywords:** psychiatry, bipolar disorder, high risk, early intervention, prodromes, psychopharmacology, psychotherapy, nutraceuticals

## Abstract

The onset of prodromal symptoms in subjects who are at familial or clinical risk for bipolar disorder could be considered as an important alarm bell for the development of the disease and should be carefully detected. The management of prodromes in bipolar high-risk patients appears to be an important means of prevention; nevertheless, at the moment, there aren’t clear and widely shared treatment indications. The aim of this review is to summarize the available treatment options (pharmacological, psychosocial and nutraceutical) for the management of prodromal symptoms in subjects who are at familial or clinical risk for bipolar disorder.

## 1. Introduction

Bipolar disorder (BD) is a severe mental illness that usually begins in the early ages, and its course is associated with high levels of functional impairment and poor quality of life [1].

The high-risk status for the development of bipolar disorder has been defined by familial criteria—the presence of at least one first- or second-degree relative with a lifetime history of bipolar I or II disorder [2]—or clinical criteria—the presence of subsyndromal affective episodes that do not meet full DSM-5 criteria for bipolar I/II disorder for duration and severity [3,4]. Youth who have familial risk for BD and present depression, anxiety, mood instability and subthreshold manic symptoms have a 49% of risk of converting to BD [2,5]. Recent evidence hypothesized BD as a progressive disease, whose trajectory starts from aspecific non-mood symptoms, such as those of childhood anxiety disorders, and continues with depression or subthreshold hypomanic episodes in adolescence and early adulthood, up to the onset of first full mood episode [6,7].

Prodromal symptoms frequently appear prior to the index affective episode. Among them, the strongest predictors of new onset of the disorder are depressive symptoms [8], anxiety [9,10], sleep disturbances [11] and subthreshold hypomanic symptoms [2,12,13,14,15,16]. The identification of these prodromal symptoms is important to play out early intervention strategies that could prevent or delay progression of BD, decreasing the total time of illness and mitigating neuroplasticity adverse effects due to repeated mood episodes [17,18].

Furthermore, planification of an early intervention is of particular importance to reduce the time interval in the absence of adequate treatment. The time interval between the onset of the prodromal phase of the disorder and the initiation of proper treatment is defined as duration of untreated illness (DUI), and it is associated with a worse course of the disease, poor level of remission and social functioning, and greater relapse rates and suicide attempts (Ienciu et al., 2010; Buoli et al., 2020) [19,20].

Literature on this topic is still lacking and often provides conflicting results. There are wide debates on the utility of starting a treatment prior to the onset of a full-blown disorder in subjects who may not develop the disease in the future. Possible options for the treatment of subthreshold bipolar symptoms include pharmacological treatment, psychosocial intervention, and nutraceutical intervention.

No guidelines are available for administering preventive psychopharmacological medication for the high-risk population [10,21,22,23]. Each pharmacological treatment is burdened by a series of adverse events and risks, which, in some circumstances, outweighs the benefits. Pharmacological approaches in at-risk populations have often shown a lack of real efficacy [24]. Nevertheless, some compounds used in the treatment of bipolar disorder are also widely used for the management of the above-mentioned symptoms in clinical practice [25].

Psychotherapy seems to be the most suitable intervention in subjects who have not already manifested a real mood episode, due to minimal adverse effects. Psychological therapies have effect on several brain regions involved in the pathogenesis of bipolar disorder, such as the amygdala, insula and anterior cingulate [26]. Psychoeducation and cognitive-behavioral therapy, which focus on providing psychoeducation on symptoms, symptom management and cognitive regulation, are the most studied psychosocial interventions in this population. Some of these psychotherapy models have been modified and adapted to the needs of specific at-risk youth and adolescents. One example is Interpersonal and Social Rhythm Therapy (IPSRT), which directly targets specific sleep disturbances such as dysregulation of circadian patterns and total sleep time [27,28]. Another method is mindfulness-based cognitive therapy for children (MBCT-C), a specifical intervention for the management of anxiety symptoms and emotional regulation in children who are at risk for bipolar disorder [29]. The most recent systematic review exploring psychosocial interventions in ‘at risk’ populations was updated until 2019 and highlighted a variety of positive outcomes in the decrease of symptoms [30].

Nutritional supplements are a suitable option for primary prevention because of the relative lack of adverse events. Even if in several cases the efficacy of these compounds appeared inconsistent, some nutraceutical agents have shown promising results (i.e., fatty acids and N-acetyl cysteine for depression, amino acid drinks and folic acid for mania).

Two recent reviews gather the available nutraceutical interventions in the treatment of BD for analysis. They both evaluate the current evidence on the efficacy of improving symptoms by adding nutraceutical agents to psychopharmacological drugs, including long chain polyunsaturated fatty acids (PUFAs), antioxidant agents such as N-acetylcysteine (NAC), amino-acid adjunction or depletion, melatonergic compounds, vitamins such as folic acid, probiotics, other compounds—such as coenzyme Q10—involved in mitochondrial biogenesis, and combination of different types of nutraceuticals (Fusar-Poli et al., 2019) [31,32].

The aim of the present review is to collect and summarize the available treatment options (pharmacological, psychosocial and nutraceutical) for the management of prodromal symptoms in subjects who are at familial or clinical risk for BD.

## 2. Methods

In November 2020, an electronic search on PubMed about prodromal symptoms of bipolar disorder, without any filter or MESH restriction, was performed using the following search strings: “at-risk” OR “high risk” AND “bipolar” AND “prodromes” OR “prodromal symptoms” OR “depression” OR “hypomanic symptoms” OR “anxious symptoms” OR “sleep disturbances” AND “therapy”; “intervention”; “treatment, program”.

The search was restricted to those articles published from 1998 onward; this year marked the first publication from the Canadian high-risk offspring cohort study [33]. This is the first longitudinal study assessing prodromal symptoms in offspring of patients with BD that included a large population.

All kinds of publications (i.e., reviews, original contributions and case reports) were included. Publications had to concern the treatment of prodromal symptoms of BD as the principal issue. Publications written in a language other than English were excluded.

Where data were not sufficient for a clear recommendation or were not available, we included guideline-based treatment options approved for similar symptomatology in full blown bipolar disorder that seemed useful for the planning of future research. For the scope, we decided to include in our research the most recent literature on mood episodes with mixed features, on Bipolar Disorder Not Otherwise Specified (BD-NOS) or based on pediatric bipolar disorder guidelines.

According to the PICOS model, the inclusion criteria could be described as follows: participants: subjects at clinical or familial risk for BD; interventions: psychopharmacological treatments, psychotherapies and nutraceutical agents; comparison: observation or watchful waiting; outcomes: reduction of prodromal affective symptoms and prevention of index affective episode; study design: RCTs (observational, retrospective and longitudinal open-label studies), metanalyses, reviews and case reports.

## 3. Results

**Figure 1:**
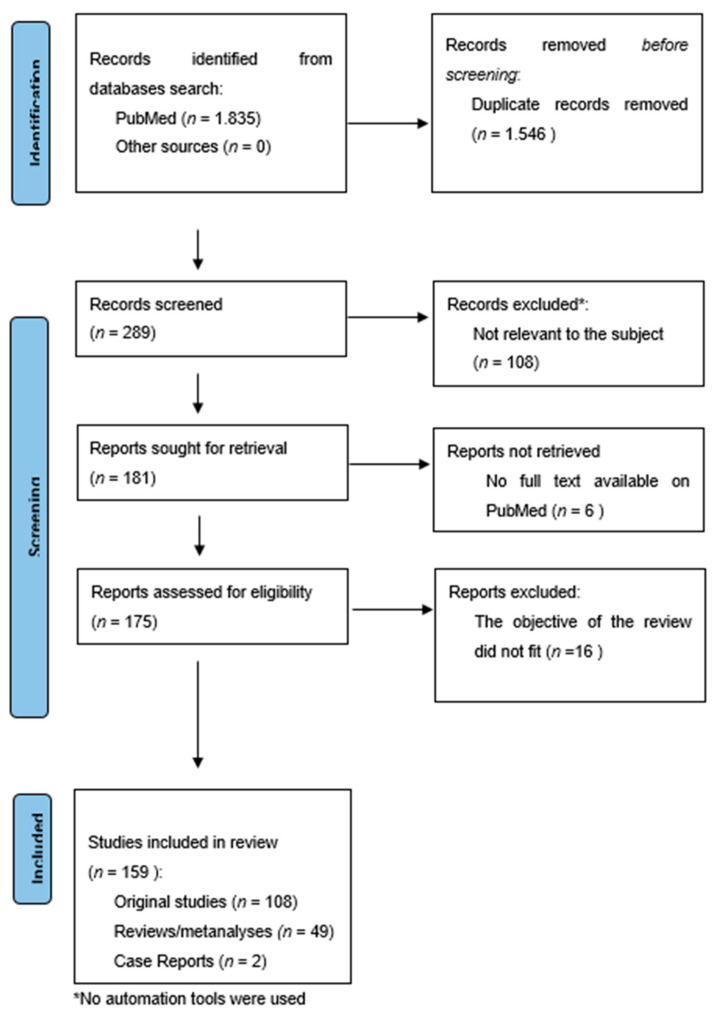
Flow chart according to the PRISMA Statement [34].

The search provided 1835 results. The overlapping studies (1546) were excluded. All references retrieved from the databases were screened based on their titles and abstracts (289). Subsequently, full texts of potentially eligible articles were further evaluated (181). All references within the included studies and those of any previous pertinent reviews were carefully reviewed to identify additional relevant studies. A total of 16 were excluded because they did not fit the objective of the review and six were excluded because the complete manuscript was not available as full text on PubMed. The final number of records included was 159. Among these, 108 were original studies (RCTs, longitudinal open-label studies, and retrospective studies), while 49 were reviews/meta-analyses and two were case reports. Figure 1.

### 3.1. Depressive Symptoms

The most common prodromal sign of mood disorder is the onset of subthreshold depressive symptoms.

Subthreshold depressive symptoms could be defined as depressed mood or loss of interest or pleasure plus at least two of the DSM-V criteria for Major Depressive Episode over a period of at least one week. The severity of these does not reach the criteria for Major Depressive Disorder and does not have a severe impact on global functioning [24].

Retrospective studies on adolescents who have developed BD indicate that the majority have presented at least subthreshold depressive symptoms before the onset of the disorder (up to 50–80%), with around 20–30% presenting atypical features (hypersomnia, hyperphagia, irritability, seasonal patterns and psychomotor slowing) [8,35].

Nevertheless, no clear guidelines are actually available for the treatment of the at-risk population.

#### 3.1.1. Pharmacological Treatment

A few studies have evaluated the efficacy of pharmacological interventions for the treatment of depression in youth at risk for BD. The results of RCT regarding compounds currently used for bipolar disorder (mood stabilizers and antidepressants) have provided mixed results in this population.

##### Mood Stabilizers

Three studies have examined the effectiveness of valproic acid in children at familial risk for BD. The first one [36] showed “much” or “very much” improvement in clinical symptoms, evaluated with Clinical Global Impression-Improvement (CGI-I), while the other two RCTs [37,38] showed no particular effectiveness regarding severity of depressive symptoms in youths treated with valproate. In addition, when valproate was combined with paroxetine [38], the onset of manic symptoms or suicidality in over half of the nine patients enrolled caused the discontinuation of the study.

The efficacy of lithium on depressed offspring of BD-affected parents was highlighted in a review that showed lithium may be well tolerated and effective in reducing suicidal ideation and depressive symptoms [39]. In agreement with these results, Nogueira-Lima [40] reported a single child case-report in which lithium monotherapy, in addition to parental psychoeducation, improved depressive symptoms and suicidal behavior.

On the other hand, a six-week, double-blind, placebo-controlled RCT was performed in 30 prepubescent depressed children with family history predictors of BD to evaluate the efficacy of lithium [41]. In this trial, no differences in depressive symptoms were found between the placebo and lithium-treated group, but some of participants dropped out of the study due to adverse effects after lithium consumption.

##### Antipsychotics

Quetiapine was evaluated in only one single-blind study. A sample of 15 adolescent patients, treated with a mean of 460 mg/daily of quetiapine, showed statistically significant improvement in both manic and depressive symptoms and showed low side effects (moderate weight gain, no sedation or other adverse events) [42].

Even if lurasidone has not been approved for the treatment of depressive symptoms in high-risk populations, its good profile of efficacy and tolerability in BD patients could make it an interesting option. Lurasidone-induced mania has been reported infrequently, and it is mostly related to lower doses. In fact, a lower dosage of lurasidone (20 to 60 mg/day) is associated with a greater serotoninergic effect that could be risky in BD, while at higher dosages (80–120 mg/day), antidopaminergic effects are prevalent [43,44].

##### Antidepressants

The Canadian Network for Mood and Anxiety Treatments (CANMAT) and the International Society for Bipolar Disorders (ISBD) 2018 guidelines for the management of patients with bipolar disorder present strong advice against the use of antidepressants in monotherapy in the treatment of bipolar depression and recommend adequate education of patients and caregivers regarding early warning symptoms of mood switching [25]. No antidepressants are approved for at-risk stages of BD.

The Food and Drug Administration (FDA) has approved certain antidepressants for the treatment of major depressive disorder in children and teenagers: fluoxetine up to eight years old and escitalopram up to age of 12. An olanzapine and fluoxetine combination is the only pharmacological treatment approved for bipolar depression in children and adolescents. Nevertheless, its use is burdened by a significant weight gain and development of metabolic symptoms. It has not been tested in a high-risk population [45]. Antidepressants, especially Selective Serotonin Reuptake Inhibitors (SSRIs), are widely used, after or in combination with psychotherapy, for the treatment of depressive symptoms in youth, regardless of family history. No clear efficacy and safety have been demonstrated regarding the use of antidepressants in the case of prodromes of BD. Their use is associated with the risk of anticipating the development of a full-blown illness, while the efficacy on depressive symptoms seems to be lower than in major depressive disorder [37]. In children at high risk for BD who present hyperactivation, antidepressants could result in the worsening of behavioral problems, impulsivity and aggressivity as well as a greater probability of medication discontinuation [46].

The rate of antidepressant-induced activation is nearly 50% in children and youth at high risk for BD [46], while a retrospective study found that up to 83% of patients that switched to mania were previously treated with SSRIs [47]. This increased risk of conversion was confirmed in a recent naturalistic RCT that included 93 bipolar offspring with anxiety or depressive symptoms treated with antidepressants [48]. In this work, 68% of at-risk patients who met full criteria for major depressive disorder, hypomania or mania, had been previously treated with antidepressants. The odds ratio for switching to mania associated with antidepressant use during follow-up was 7.9.

If an antidepressant treatment is needed due to severity of symptoms, it should be cautiously selected and titrated and should include careful monitoring of any treatment-emergent adverse events. Tricyclics are less tolerated than SSRIs [49], and higher rates of switching to mania are associated with nortriptyline [50]. Yamaguchi presented a series of clinical cases that demonstrate the relative better safety profile of escitalopram among SSRIs. Escitalopram-induced mania is most likely linked to an higher dosage (20 mg/daily or more) and appears shortly after reaching the maximum dosage, so it can be easily monitored [51]. Apart from escitalopram, fluoxetine has the best efficacy/safety profile and is approved for use in children and adolescents [52], while venlafaxine is associated with a high risk of switching to mania [49].

#### 3.1.2. Psychosocial Interventions

International guidelines for BD identify psychotherapy as the first line treatment for depressive symptoms for BD, in addiction to pharmacotherapy. Available literature about psychosocial therapy for depressive symptoms in people at risk of BD includes three studies about Family Focused Therapy (FFT) [23,53,54] and one study that evaluates the efficacy of cognitive-behavioral (CBT) group therapy [24]. Preliminary results about psychoeducation are available.

##### Family Focused Therapy

FFT is a psychoeducation family intervention that enhances communication and problem-solving strategies to reduce intrafamilial stress, conflict and affective arousal as criticism, hostility or emotional over-involvement in caregivers. FFT has already shown good results for schizophrenia [55], major depression, prodromal psychosis, and bipolar I and II illness [54,56,57].

In 2011, Miklowitz and colleagues designed an adapted FFT for family of children at high risk for BD (FFT-high risk version: FFT-HR) that has as its principal aim to assist the youth and family members in recognition and acceptance of early signs of prodromal symptoms and prevention of mood escalation. In addition to pharmacotherapy as needed, FFT-HR has demonstrated better efficacy than educational control in preventing, delaying and reducing depressive symptoms (significant improvements in Children’s Depression Rating Scale) [53]. Two other subsequent randomized trials conducted by the same authors confirm that FFT in high-risk populations, in combination with pharmacotherapy, resulted in effective mitigation of mood symptoms prior to full BD as well as delaying recurrences and improving global functioning at the early phases of the illness [23,54].

##### Cognitive-Behavioral Therapy

CBT is recommended by the international guidelines for both high-risk patients for psychosis and for early stages of BD due to its effectiveness in reducing symptomatology and its lower incidence of adverse events [58]. In 2020, an early specific CBT (“Cognitive-behavioral therapy applied early in the potential developmental course of bipolar disorder—EarlyCBT”) was proposed to at-risk patients for BD to evaluate its efficacy and safety [24].

The theory of this model was adapted on the basis of the principles for stress management and problem solving described in previous works “BEsT (be)for(e) Bipolar” by C. Marx, Leopold and Pfennig, 2009, “Cognitive behavioral treatment manual for bipolar disorders” by Meyer and Hautzinger [59] and a psychotherapy model described by Bechdolf and Juckel [60]. The sessions included psychoeducation about BD, early warning signs and crisis management and offer cognitive strategies for prevention of and sensitization to mood episodes. The CBT group was compared to a control group that received non-directive psychotherapy. The results were controversial. In fact, the whole sample manifested an improvement in depressive symptoms, but no differences were found between the CBT group and the control group in the Hamilton Rating Scale for Depression (HAMD) and the Bipolar Prodrome Symptom Scale-Prospective (BPSS-P).

##### Psychoeducation

A recent review evidenced the efficacy of psychoeducation in reducing global symptom severity (one study reported), depressive symptom severity (three studies reported) and manic symptom severity (five studies reported), especially in BD patients with a lower number of previous mood episodes (less than 10 episodes) [61]. Thanks to promising results, more studies should be carried out on psychoeducation as a possible prevention strategy, even in at-risk populations.

#### 3.1.3. Nutraceuticals

Nutraceutical compounds that have been studied for the relief of depressive symptoms in psychiatric conditions include long chain polyunsaturated fatty acids, antioxidant agents (such as N-acetylcysteine (NAC)), folic acid and compounds (such as coenzyme Q10) involved in mitochondrial biogenesis [31,32].

##### Polyunsaturated Fatty Acids

In the last few decades, the role of Polyunsaturated Fatty Acids (PUFAs) in the prevention of psychiatric disorders has received significant attention. Omega-3 PUFAs take part in neuronal growth, development and function, acting as a neurotrophic factor [62]. EPA and DHA are the major components of omega-3 fatty acids. They seem to play a role in reducing the neuroinflammation and autoimmune effects linked to the development of depressive symptoms [63]. Severe n-3 PUFA deficiency causes an imbalance in dopaminergic and serotonergic pathways, which could be linked to symptoms such as lack of motivation, decreased response to reward, and impairment in intellectual abilities [64]. Moreover, lower levels of EPA and DHA in peripheral tissues (plasma, serum, and red blood cells) are common in subjects with a diagnosis of depressive or bipolar disorder [65,66]. A series of reviews [62,67,68] and meta-analyses [69,70] investigated the efficacy and tolerability of omega-3 fatty acids (DHA and EPA) on depressive symptoms in major depressive disorder, BD and high-risk states for psychosis, while no data could be found about high-risk conditions to develop BD. The most recent review [62] reported good results in major depressive disorder treated with supplementation of n-3 PUFAs (ranging from 0.4 to 6.2 g/day of EPA and from 0.2 to 3.4 g/day of DHA) in combination with antidepressants. Regarding BD, two studies and four reviews showed an improvement of depressive symptoms in patients treated with a combination of mood stabilizers and supplementation of PUFAs. While another recent metanalysis [71], which included 31 RCTs on the effects of omega-3, omega-6 or total PUFAs on depression and anxiety, showed little or no effect in terms of prevention against depressive or anxiety symptoms in BD.

##### N-acetylcysteine

NAC is a precursor of endogenous antioxidant glutathione and has antioxidant, anti-inflammatory, and neuroprotective properties [72]. No studies about the use of NAC on high-risk patients for BD have been carried out, but it has previously been investigated in the prevention and treatment of psychosis [73] and in the treatment of depressive disorder and BD, showing heterogeneous effects in a clinical setting.

##### Folic Acid

Folate assumption has been studied specifically for the prevention of mood episodes in a familial risk population for BD.

A meta-analysis showed a significant relationship between low folate levels and risk for depression [74]. Folate deficiency is associated with accumulation of homocysteine, which, in excess, can promote nerve cell death. In a randomized, double-blind, placebo-controlled trial, Sharpley and colleagues [75] hypothesized that folic acid supplementation given to young people at increased familial risk of mood disorder may convey a neuroprotective effect and could prevent the first mood episodes. A dosage of 2.5 mg daily showed mild evidence that folate may delay the onset of an episode of mood disorder and make it clinically milder.

##### Others

Positive results have been associated with use of coenzyme Q10 on depressive symptoms in BD. Two studies [76,77] showed an improvement in depressive symptoms and functioning in BD patients at the end of the treatment (respectively 8 and 20 weeks after the beginning of the treatment). The efficacy, the good tolerance and lack of adverse effects could make coenzyme Q10 an interesting option for future studies in high-risk BD populations.

### 3.2. Anxious Symptoms

Anxiety symptoms occur early and are frequent in youth who have a familial history of BD. In a prospective study, Duffy and colleagues showed that cumulative incidence of anxiety disorders was almost 15% higher and occurred earlier in youth at high risk for BD compared with controls from families with no history of psychiatric illness [78]. Anxiety symptoms may precede or co-occur with depressive and sub-syndromic hypomanic symptoms and are associated with future poor treatment response. Especially at the onset, anxious prodromes in patients at high risk of developing BD might be underestimated and often are treated exclusively with symptomatic drugs [16,79].

Neuroanatomical studies supported by functional magnetic resonance imaging (fMRI) found in a greater amygdala excitability [80,81,82] the basis of the development of anxiety symptoms. The same exaggerated excitability is also common in subjects that have a diagnosis of BD or anxiety disorder [83,84]. In particular, HTTLPR genotypes with s-allele carriers manifest both a greater amygdala excitability and increased anxiety-related temperament traits [85,86,87,88,89].

#### 3.2.1. Pharmacological Treatment

The latest guidelines on BD [25] from the International Society for Bipolar Disorders give an indication on how to treat anxiety that co-occurs with BD but do not provide recommendations for at-risk populations.

##### Benzodiazepines

Benzodiazepines (BZDs) provide fast relief in the reduction of comorbid anxiety and are approved by Canadian Network for Mood and Anxiety Treatments as symptomatic drugs in the short-term treatment in BD; however, the appropriateness of their use in at-risk patients for BD is debated. Two recent works [79,90] asserted that prolonged use of BZDs in at-risk populations may extend the duration of untreated illness up to four years, delaying the administration of an adequate psychopharmacological treatment and an appropriate medical follow-up. The risk of drug misuse and dependence in BD patients appears higher than in other patients. The yearly incidence of dependence and abuse reaches 6.3% in those using clonazepam, alprazolam, and multiple benzodiazepines alone or in combination with Z-drug treatments [91]. Thus, BZDs should be prescribed at the lowest possible dose for the shortest period possible, with a maximum of four weeks. Furthermore, BZD users having BD are more vulnerable to develop paradox effects, such as harmful depressogenic effects [92,93], worsening anxiety symptoms and rapid mood relapses [85].

##### Antidepressants

As previously discussed, antidepressants, which are commonly used to treat anxiety disorders in the young, are poorly tolerated in at-risk BD patients and may accelerate the onset of mania [46]. Studies on children and adolescents with depression or anxiety disorders have reported high rates of agitation or anxiety-like symptoms in offspring of BD parents during antidepressant treatment [85]. The introduction of antidepressants should be limited to more severe cases and should be carefully monitored for emergence of agitation or manic symptoms.

##### Mood Stabilizers

Mood stabilization accompanies the reduction of anxious symptoms in co-occurring anxiety and BD. Valproic acid, carbamazepine, quetiapine and olanzapine may have specific anxiolytic properties. However, not all of these have been tested in at-risk populations and some are associated with important side effects. A familial history of anxiety disorder is a predictor of response to lamotrigine in patients with BD [94,95]; thus, it could be an interesting field for future trials.

##### Others

Pregabalin is effective, well tolerated and is not associated with a risk of mood destabilization. It has not been tested in BD with co-morbid anxiety, but it could be an appropriate option in clinical practice [25].

#### 3.2.2. Psychosocial Interventions

##### Cognitive-Behavioral Therapy

CBT is the first line of intervention for the management of anxiety in children and adolescents with mood disorders [25,46]. Common CBT models, which have shown effectiveness for depressive symptoms, seem to be also effective for anxiety symptoms [61] in young patients with BD.

Mindfulness-based cognitive therapy for children (MBCT-C) is a manualized group psychotherapeutic intervention proposed by Cotton and colleagues for young patients at familial risk for BD, with the aim of enhancing attention and emotion regulation processes. It consists of 12 sessions that incorporate cognitive behavioral principles and mindfulness skills based on acceptance and non-judgmental awareness of thoughts [29]. MBCT-C was associated with improvements in clinician- and self-rated anxiety (seven points means improvement on a symptom scale) and emotional regulation [18,96,97].

##### Nutraceuticals

Among nutraceutical agents, only PUFAs have been investigated in the management of anxiety, though they have not been tested for anxiety that occurs in patients with clinical or familial risk for the development of BD. Thanks to promising results among people with mental disorders, PUFAs could be an interesting option for the reduction of anxious symptoms, even in at-risk populations. Observational research suggests correlations between low omega-3 and a high omega-6/omega-3 ratio—in particular, eicosapentaenoic acid (EPA) and docosahexaenoic acid (DHA)—and both depression [98,99] and anxiety scores [100]. A Spanish cohort study that included 7903 participants with a prospective two-year follow-up analysis suggested an initial beneficial effect of moderate intake of omega-3 fatty acids (a mean of 83.3–112 g/day) on comorbid anxiety [101]; the result was not confirmed in the final models. Three recent randomized clinical trials [102,103] reported an inverse association between high doses of DHA and EPA (ranging from 300 to 3360 mg/d of EPA plus DHA) and the severity of anxiety symptoms and anger.

### 3.3. Sleep Disturbances

Circadian rhythms are strictly linked to the regulation of affective states [104]. Sleep disturbances, including variability in sleep duration, excessive daytime sleepiness, sleep fragmentation, impaired sleep efficiency, and REM sleep disturbances, are common in offspring of parents with BD [28,105,106]. Sleep deprivation and insomnia are linked to alteration in functional connectivity between the medial prefrontal cortex and the amygdala during reward processing. Neuroimaging studies, performed by fMRI, found that the same areas show a dysfunctional activation in patients with BD [107,108].

Studies of EEG patterns have shown that people with an elevated risk of developing BD have weaker sleep efficacy and mean lower total sleep time in comparison with controls [109]. Recent studies have noted an association between sleep, motor activity, energy and mood in people who suffer from BD [110]. Alteration of the baseline circadian rhythm could be considered as a prodrome of the onset of both depressive and maniac episodes [11,108]. Poor-sleeping high-risk youth are more vulnerable to future BD compared to good sleepers. Furthermore, circadian rhythm dysregulation increases cortisol secretion and inflammatory activity, which play a role in the conversion to BD and in the severity of mood alterations [111,112,113].

#### 3.3.1. Pharmacological Treatment

##### Benzodiazepines and Hypnotics

Pharmacological treatment of sleep symptoms usually includes early adoption of hypnotic BZDs and hypnotics. Therefore, as mentioned above [91], these drugs are associated with several adverse effects in high-risk BD populations. In addition, BD has high association rates with pharmacophilia and substance misuse. Given the risk of abuse in this population, certain classes of FDA-approved insomnia medications, such as BZDs and hypnotics, should be avoided [114,115].

##### Sleep-Promoting Antidepressants

Sleep-promoting antidepressants (such as mirtazapine, trazodone and agomelatine) are commonly used in the treatment of insomnia as an alternative to BZDs and non-benzodiazepine hypnotics, thanks to the possibility of being used for a prolonged time and the low risk of dependence.

At the moment, data about the efficacy and safety of sleep-promoting antidepressants in high-risk people for BD are insufficient.

The literature regarding their use in BD raises concern about the risk of switching to mania. In 2015, Wichiniak and colleagues collected a series of clinical cases about the risk of switching to mania associated with trazodone, mirtazapine, and agomelatine. In agreement with previous works [116,117], the risk of switching was confirmed but seemed to be mostly associated with an early stage of treatment and a higher dosage (drugs were used at an antidepressant dosage); no cases were reported in the case of association with concomitant mood-stabilizer therapy. On the other hand, trazodone, mirtazapine and agomelatine provide hypnotic or sedative effects even at low dosages (up to 100 mg/day for trazodone, 3.75–15 mg/day for mirtazapine and up to 25 mg/day for agomelatine), which seem to be safe in patients with BD [118].

#### 3.3.2. Psychosocial Interventions

There is a reasonable level of evidence that sleep-related interventions could be beneficial in the management of prodromal sleep disorders that occur at the early phases of mood episodes. Intervention such as Interpersonal and Social Rhythm Therapy (IPSRT) and CBT have been evaluated even in at-risk populations.

##### Interpersonal and Social Rhythm Therapy

IPSRT helps to stabilize the sleep-wake rhythm and daily routine and has provided good results in delaying BD recurrence in adults. In 2014, Goldstein conducted an open trial of IPSRT, planned on the basis of “IPSRT—A treatment manual for youth with BD” [119]. The intervention was targeted at adolescents at risk for BD (decreased treatment length, interpersonal problem session focused on the adolescent’s feelings about having a parent or sibling with BD).

Data from the pilot study suggested a significant improvement in sleep patterns, with less oversleeping, but minimal or no difference in preventing or improving mood symptoms. In addition, the low numerosity of the initial sample (thirteen adolescents) and non-completion of the whole program by many of the participants constituted important limits of this study [27]. In 2018, the same study group conducted a pilot randomized trial of IPSRT plus data-informed referral (DIR), a referral for psychiatric community treatment, versus DIR alone for adolescents with familial risk of BD. The 42 adolescents in the study had a BP parent and had to wear an actigraph to measure sleep/wake patterns for seven days at baseline and at six months. Participants that received IPSRT + DIR, as compared with DIR alone, were significantly improved regarding continuity of sleep. The better quality of sleep was also linked to a reduction in manic and hypomanic subthreshold symptoms [28].

##### Cognitive Behavioral Therapy

The efficacy of cognitive behavior therapy for insomnia (CBT-I) is supported by multiple meta-analyses and works [114,120]. The American College of Physicians recommend it as first line of treatment prior to pharmacotherapy [121] in both short-term and long-term perspectives on BD [122]. The core of cognitive behavior therapy for insomnia (CBT-I) includes several sessions focused on sleep restriction therapy, psychoeducation about sleep, stimulus control and perception of sleep. CBT-I for BD has not been evaluated in at-risk populations, but results demonstrate an improvement in sleep and circadian disturbance and also show benefits in mood symptoms during the treatment and during the follow-up period [123]. CBT-I for BD includes elements of IPSRT [124], Motivational Interviewing and Chronotherapy [125] and has a particular focus on correcting sleep state misperception [122]. Three trial protocols evaluating CBT-I in BD [122,126] and in multiple psychiatric disorders [123] are ongoing.

##### Chronotherapeutic Intervention

Chronotherapeutic intervention is a recognized treatment for bipolar depression based on the effect of light and dark and on the control of sleeping times on the regularization of wake-sleep rhythm. It includes bright light therapy (LT), dark therapy (DT), treatments utilizing sleep deprivation (SD), melatonergic agonists (MA), interpersonal social rhythm therapy (IPSRT), and cognitive behavioral therapy adapted for BD (CBTI-BP). Even though no results are available for its use in at-risk populations, it has provided good results in improving mood symptoms and sleep disturbances associated with BD, [114,127] and it seems safe even in children and adolescents. All chronotherapeutic interventions seem to prevent the relapse of maniac episodes via regularizing the dysregulated sleep patterns that appear early in the development of a mood episode [128]; thus, it could be an interesting option in the prevention of the illness. The International Society of Bipolar Disorders (ISBD) task force on chronobiology and chronotherapy recently published a systematic review of the literature about the use of the five classes of chronotherapy [126]. This work presents important limitations due to the heterogeneity of methods and objectives of the papers included. Nevertheless, the sum of the results suggests that LT and SD are both efficacious in the relief from depressive symptoms, cuing circadian rhythm, improving subjective sleep quality and decreasing excessive sleep in depressive phases of BD [114,127]. The results are concordant with a previous metanalysis that showed that LT reduces disease severity by regularizing the circadian rhythm [129]. DT as a blue-blocking regime could rapidly block the escalation of sleep symptoms associated with mania, acting on reduction in total sleep, reduction in motor activity during sleep intervals, and increased regularity of sleep intervals [126].

#### 3.3.3. Nutraceuticals

##### Melatonin

Melatonin is a neurohormone that is synthesized by the pineal gland during dark periods and regularizes the sleep-wake rhythm [108]. Immediate-release melatonin supplements have provided solid evidence in regularizing circadian rhythm alterations [130], in combination or without BZD assumption. Melatonin elicited an increasing interest as a result of neurobiological studies that found a higher suppression of melatonin in response to light in patients with BD [131]. Studies have shown that patients with BD exhibit a relative lack of endogenous melatonin due to impaired activity of the enzyme that converts serotonin into melatonin (acetyl serotonin O-methyltransferase—ASMT). Even in patients at risk for BD, ASMT showed several mutations that cause imbalances in melatonin levels and in the expression of melatonin receptors in the central nervous system [113,132,133,134].

RCTs regarding the use of melatonin in the treatment of sleep disorders in BD are lacking and heterogeneous; no studies have been conducted on high-risk subjects for BD. Further studies and larger samples are needed to make clear recommendations on the use of melatonin receptor agonists in sleep disturbances in patients at risk of BD or in the early phases of BD [135].

### 3.4. Hypomanic Symptoms

Subthreshold manic symptoms are less common than depressive symptoms but are pathognomonic for BD. Subthreshold mania is defined as a period of at least two consecutive days of abnormally and persistently elevated, expansive or irritable mood plus at least two of the following criteria: inflated self-esteem or grandiosity, decreased need for sleep, more talkativeness than usual, flight or rapid racing of ideas, distractibility, increased goal-directed activity, or psychomotor agitation. The symptoms are milder and do not reach the full criteria for a manic or hypomanic episodes [24].

An alternative definition of subsyndromal hypomanic features indicates the presence of two or more symptoms that reach a severity score of two or more on the Young Mania Rating Scale [136,137]. The onset of subthreshold hypomanic symptoms is considered by some authors as the beginning of full-blown BD, and it is diagnosed as Bipolar Disorder Not Otherwise Specified (BP-NOS).

Two recent naturalistic prospective studies report that between one-quarter and one-half of children and adolescents who present hypomanic symptoms and have a family history of mania convert to full-blown BD over 5–8 years [2,14]. The Pittsburgh bipolar offspring study [5] and several subsequent studies [16,24] confirmed that subclinical manic symptoms were the stronger predictor for subsequent hypomania and mania onset, especially in early ages. In addition to depressive symptoms and changes in sleep patterns, they are considered as the main risk criteria in all published early recognition assessments for BD [22,138].

Manic and hypomanic symptoms, in particular in younger subjects, could be mistaken for other psychiatric manifestations such as anxiety or behavior disorders in children. The initial prodrome appears for a sufficiently long time for encouraging early identification and planning adequate interventions, especially in youth who have a familial risk for BD and have not yet developed the disease [139].

#### 3.4.1. Pharmacological Treatments

The need of pharmacological treatment for hypomanic subthreshold symptoms for the prevention of the onset of BD in the at-risk stage is still debating. The long- and short-term tolerability of each treatment needs to be carefully weighed against the individual risk of developing the illness.

In practical scenarios, high-risk symptomatic youth tend to be treated with a wide variety of medications or do not receive any treatment at all [22,140,141]. Nevertheless, the delay in starting an adequate treatment can lead to a major severity of the disease and less time in the euthymic phase in adulthood [21].

Few studies focus on the pharmacological management of hypomanic symptoms in high-risk patients. Some pilot studies have assessed the preventive effect of valproic acid and quetiapine against the onset of mania, but the results are mixed [35,37,42] and the presence of short- and long-term adverse effects make their use not recommended [142]. Nevertheless, in the case of more severe hypomanic symptoms (that increase the risk of conversion to manic or hypomanic episodes), the beginning of a pharmacological treatment could be considered more appropriate [138,143]. No pharmacological option has been approved for the treatment of at-risk patients, so possible options for the treatment of more severe subthreshold manic symptoms could be evaluated on the basis of available treatment for adolescent and pediatric mania [140] or the treatment of depressive episodes with mixed features.

In children and adolescents, atypical antipsychotics (risperidone, olanzapine, quetiapine, aripiprazole, ziprasidone, asenapine; [144] Findlings et al., 2018) are approved by the United States Food and Drug Administration (FDA) as first-line treatment in the management of hypomania and mania [28]. They have appeared to be superior in the reduction of manic symptoms to lithium—which has demonstrated some efficacy only in the case where there is a family history of a positive lithium response [140,145,146]—valproate and carbamazepine, when used as monotherapy [147,148,149].

Mixed features that occur in depressive episodes could be considered similar to subthreshold hypomanic symptoms (such as increased activity, mood instability and agitation). They are considered as risk factors for conversion in high-risk patients and are common in depressive episodes in BD [138,150]; thus, their treatment could provide possible options for subthreshold hypomanic symptoms in high-risk states.

Regarding treatment of mixed features in manic and depressive episodes, aripiprazole, lurasidone, asenapine, carbamazepine, olanzapine (as monotherapy and in combination with lithium or valproate), and ziprasidone produced the strongest evidence of efficacy [150]. Among them, aripiprazole and lurasidone are of particular interest for high-risk states due to their low risk of side effects.

In a double-blind placebo-controlled trial in 59 children and adolescents at familial risk for BD, Findling and colleagues [144] demonstrated that aripiprazole (mean dosage of 7.1–7.4 mg/day) causes a significant and rapid reduction of manic symptoms (evaluated by YMRS and Clinical Global Assessment-Severity; CGI-S) and a global improvement in functioning (assessed by the Children’s Global Assessment Scale; CGAS). During the 12 weeks of the trial, the rate of adverse events was low, and participants reported good satisfaction regarding this treatment. Five short-term RCTs demonstrated that aripiprazole is efficacious in reducing manic symptoms in depressive episodes with mixed features and preventing recurrences of manic and mixed episodes. It also appeared safe and well tolerated even in children and adolescents [37,151,152,153]. Lurasidone has provided excellent results in the treatment of affective episodes with mixed features, especially in early the phases of BD, improving the severity of both depressive and manic symptoms [137,154,155,156] and showing a good safety profile.

#### 3.4.2. Psychosocial Interventions

Psychosocial interventions seem to have fewer positive results on hypomanic symptoms than on depressive and anxious symptoms.

##### Cognitive Behavioral Treatment

The Early CBT model adapted to at-risk patients (“cognitive-behavioral therapy applied early in the potential developmental course of bipolar disorder”), developed by Leopold and colleagues [24], has been proposed for at-risk patients for BD for the prevention and management of early hypomanic subthreshold symptoms. Final results were moderate: the severity of manic symptomatology at YMRS was slightly but significantly decreased in the intervention group, while no significant difference was found in the change of manic features, evaluated by the Bipolar Prodrome Symptom Scale-Prospective (BPSS-P), between the control and treatment group [24]. The literature about the efficacy of CBT on hypomanic symptoms in BD (at-risk and full-blown BD) provides scarce results. In 2016, Yu Ye completed a meta-analysis that found that the improvement in the Young Mania Rating Score (YMRS) during CBT rapidly decreased after three and six months of follow-up. On the other hand, all but one of the nine studies included in the meta-analysis that used YMRS [59] showed no difference between baseline and post-treatment symptoms [157].

##### Inter-Personal Rhythm Social Therapy

As mentioned above, in 2018, Goldstein performed a larger scale RCT pilot study on the evaluation of efficacy of IPRST plus data-informed referral (DIR) on a sample of youths at risk of BD [28]. Contrary to the previous work [27], in which no differences were found on preventing or improving mood symptoms, in this work, the IPRST group showed improvement not only in sleep patterns but also in the severity of subthreshold hypomania symptoms over the follow-up period. In addition, subthreshold hypomania symptoms were present for less time. This pilot RCT demonstrates that a better quality of sleep may be beneficial in delaying or preventing subthreshold hypomanic symptoms among at-risk youths, even if further studies are needed to confirm these promising results.

##### Family Focused Therapy

The use of FFT was widely investigated for both depressive and manic symptoms. Seven randomized clinical trials of adult and pediatric populations investigated the efficacy of FFT in bipolar disorder. Five of these indicated stronger effects of FFT on depressive symptoms than manic or hypomanic symptoms [17,53,54,158,159], whereas two indicated stronger effects of FFT on manic or hypomanic symptoms [160,161]. FFT seems indifferent about the reduction of the risk of conversions to BD [23].

Miklowitz et al. performed a series of randomized controlled trials using a modified version of FFT adapted on patients at high risk for BD (FFT—High Risk Version). Families of the subjects received 12 sessions of adapted FFT over four months, in combination with psychotropic medications. Regarding hypomanic symptoms that were present prior to the development of a real BD, more rapid recovery, more weeks in remission and better YMRS scores were found in patients who participated in FFT sessions [53,55]. This benefit persists after one year of follow-up. In 2020, the same working group performed an extension of previous studies with a larger sample and a longer follow-up period (average of two years). The most relevant result was the prolongation of well intervals during the maintenance phase of treatment, while the recovery from mood symptoms showed no significative improvement.

The explanation of these results can be found in an additional study exploring areas of brain activation [162], using nine participants from the above-mentioned pilot RCT on the use of FFT in populations at risk of BD [53]. In parallel to a reduction of mania scores from pre- to post-treatment, FFT determined changes in activation in the dorsolateral prefrontal cortex and decreased activation in the amygdala, which is augmented during manic states [30,162].

#### 3.4.3. Nutraceuticals

No studies based on high-risk populations for BD are actually available with regard to the effects of nutraceuticals. Regarding the use of nutraceuticals for hypomanic symptoms in BD, omega-3 fatty acids are of particularly interest because they have been studied in children and adolescent patients.

##### Omega-3 Fatty Acids

Studies on addiction of omega-3 fatty acids have shown a scarcity of results on manic symptoms in BD [62,68], and only two RCTs have reported positive results. The first one [163] found a large score decrease on the YMRS in children who displayed mixed, manic, or hypomanic symptoms and were treated with high doses of eicosapentaenoic acid (EPA) and docosahexaenoic acid (DHA) omega-3 fatty acids, while the second [164] reported significant improvements on the YMRS in adult BD treated with a supplement of 1000 mg of omega-3.

##### Amino Acids

Initial studies about the efficacy of amino acid–enriched drinks have been carried for BD patients due to promising results and the lack of side effects. In agreement with the hypothesis that imbalances in the production of catecholamines, as a precursor of dopamine, play a role in the onset of manic symptoms [165], consumption of tryptophan-depleted, tyrosine-depleted and branched-chain amino acid–enriched drinks have been shown to have rapid antimanic effects (great improvement on YMRS within 6 h after the consumption) [166,167]. Nevertheless, the reliability of these results has to be confirmed with further studies.

##### Others

Other nutraceuticals, used as additions to standard therapy, have been tested for hypomanic symptoms in full-blown BD; however, results appeared scarce or contrasting. Slight or no differences have been found for NAC assumption [33,72,166,167,168,169,170], and results have not confirmed improvements for folic acids [171].

## 4. Conclusions

The individuation and the management of prodromal symptoms has attracted interest for the prevention and planification of tailored care in psychiatry. Although clear indications and guidelines for the management of prodromal symptoms in at-risk subjects for BD are still lacking, the available literature has provided a series of positive results.

The existing literature on pharmacotherapy for individuals at high risk for BD provides little overall evidence of benefits and is not supported by sufficient evidence. Pharmacology treatment is burdened by a series of weighty side effects, so its beginning is intended for more severe symptoms and should be evaluated after an accurate assessment of the risk/benefit ratio. The risk linked to antidepressant medications in youths at high risk for developing BD suggests that alternative interventions are needed for the treatment of prodromal depression and anxiety. BZDs should also be avoided due to the risk of misuse and dependence. Low doses of trazodone and mirtazapine (up to 100 mg/day for trazodone, 3.75–15 mg/day for mirtazapine and up to 25 mg/day for agomelatine) have a good safety profile, with low rates of switching [118], and could be considered as important alternatives to hypnotics. Lurasidone and aripiprazole have evidenced a favorable safety profile; the first one has provided good results on both depressive and hypomanic subthreshold symptoms, while the second one appeared efficacious on hypomanic symptoms.

Psychotherapy is the most studied intervention for at-risk populations. Adapted models of CBT (cognitive behavior therapy for insomnia for bipolar disorder and mindfulness-based cognitive therapy for children), IPRST and psychoeducation appeared efficacious and relatively safe and seem to be the best option for high-risk states. The involvement of the whole family, provided by FFT, appeared to be of great importance; improvement in communication and emotion regulation appeared to be useful for the management of depressive, anxious and hypomanic symptoms. Conflicting results are found regarding the use of nutraceuticals. Adjuvant treatment with omega-3 and NAC have shown mild but proven positive results on depressive symptoms and anxiety; however, too few studies have been conducted on the other nutraceutical agents. The use of such supplementation does not reach evidence for recommendation in clinical practice due to scarcity of controlled studies, heterogeneity of methods and results, and important limitations (great variability in inclusion criteria, small sample size and follow-up time).

This study presents some limitations. Above all, it was not organized as a systematic review, so it was not conducted according to PRISMA guidelines. No structured tables were used due to the heterogeneity of the studies analyzed, so the results section might not appear clear. Only one database was searched, and the protocol was not pre-published.

At the moment, evidence in the literature is still insufficient for drawing up shared guidelines about the treatment of psychiatric symptoms that at-risk patients for BD have already manifested. Our review could stimulate new perspectives on research in the field, on the basis of available data. Many of the suggested treatments lack the solid evidence necessary for formal recommendations, so further and more structured studies are needed.

## Data Availability

MDPI Research Data Policies.
